# Management of *Helicobacter Pylori* Infection and Effectiveness Rates in Daily Clinical Practice in Spain: 2010–2019

**DOI:** 10.3390/antibiotics11050698

**Published:** 2022-05-20

**Authors:** Inés Ariño Pérez, Samuel J. Martínez-Domínguez, Enrique Alfaro Almajano, Patricia Carrera-Lasfuentes, Ángel Lanas

**Affiliations:** 1 Department of Gastroenterology, Obispo Polanco Hospital, 44002 Teruel, Spain; arino.ines@gmail.com; 2 Department of Gastroenterology, Lozano Blesa University Hospital, 50009 Zaragoza, Spain; kike_almajano@hotmail.com (E.A.A.); angel.lanas@gmail.com (Á.L.); 3 Aragón Health Research Institute (IIS Aragón), 50009 Zaragoza, Spain; pcarreralasfuentes@gmail.com; 4 School of Medicine, University of Zaragoza, 50009 Zaragoza, Spain; 5 CIBER Enfermedades Hepáticas y Digestivas (CIBERehd), 28029 Madrid, Spain

**Keywords:** *Helicobacter pylori*, peptic ulcer, gastric neoplasms, treatment, efficacy, management, trends, clinical practice

## Abstract

The management and effectiveness of the treatment of *Helicobacter pylori* infection are heterogeneous worldwide, despite the publication of international consensus conferences and guidelines, which have been widely available for years. The aim of the study was to describe the clinical management and the eradication rates in a region of Southern Europe (Spain). Between 2010 and 2019, we conducted a retrospective analysis of patients with *H. pylori* infection attended by gastroenterologists in two defined areas of the National Health System in Aragón. We compared the appropriateness of therapies according to guidelines, and described the effectiveness of each treatment. A total of 1644 penicillin non-allergic patients were included. The most prescribed therapy between 2010 and 2013 was the ‘classic’ triple therapy PCA (80%), whereas the ’concomitant’ therapy PCAM was chosen by 90% of the gastroenterologists in 2015. After 2016, the use of the quadruple bismuth-containing therapy in a single capsule (Pylera^®^) quickly increased, representing almost half of the overall prescriptions in 2019. Throughout the decade, adherence to guidelines was 76.4% and global efficacy was 70.7% (ITT). Triple therapies’ eradication rates were lower than 70% (ITT), whereas eradication rates with quadruple therapies achieved or were over 80% (ITT). In conclusion, despite the use of quadruple therapies and optimized treatments, the effectiveness of *H. pylori* management in daily clinical practice is far from the target of 90%.

## 1. Introduction

*Helicobacter pylori* infection affects almost half of the world’s population [1] and has a relevant role in the pathogenesis of different clinical entities, such as dyspepsia, gastric and/or duodenal ulcers and gastric neoplasms, being the main indications for treatment. *H. pylori* infection is estimated to be associated with 48.3% of gastric neoplasms in Spain (non-cardiac gastric adenocarcinoma and Non-Hodgkin´s lymphoma) [2]. These data show the importance of achieving the highest possible eradication rates.

The increase in antibiotic resistance [3] has led to a parallel change in the therapeutic recommendations of expert committees in order to improve or maintain acceptable effectiveness levels. Since 1999, five Spanish Consensus Conferences (SCCs) have been published. These SCCs have followed or preceded the guidelines established by the Maastricht consensus. However, these recommendations may experience a significant delay in being implemented in clinical practice or may not be followed by physicians [4].

The recommendations of the second SCC of 2005 did not change significantly compared to the previous one, in which the ‘classic’ triple therapy PCA (Proton Pump Inhibitor (PPI) + Clarithromycin + Amoxicillin) was the first line of treatment [5]. The recommended duration of treatment was 7 days in the case of peptic ulcer disease and 10 days in the case of dyspepsia. Quadruple therapies were reserved as rescue treatment after the failure of first-line therapy. However, the non-bismuth quadruple therapy PCAM (PPI + Clarithromycin + Amoxicillin + Metronidazole), also called ‘concomitant’ treatment, became the first-line treatment after the publication of the third SCC in 2013, extending the recommended duration to 10 days [6]. PCA was only recommended as a first-line therapy in geographic areas with effectiveness >80%. Other therapies, such as PLA (PPI + Levofloxacin + Amoxicillin) and the bismuth-containing quadruple therapy PBMT (PPI + Bismuth + Metronidazole + Tetracycline), were suggested as second-line treatments. These recommendations were aligned with those reported by the Maastricht consensus group.

PCAM, PBMT or PPI + Pylera^®^(PPylera^®^) were placed as first-line treatment by the fourth SCC in 2016 [7]. First-line therapy not used in the first instance or PLAB (PPI + Levofloxacin + Amoxicillin + Bismuth) were proposed as second-line therapy, choosing as the third line the therapy not used in the first or second lines. The fifth and most recent SCC recommendations maintained this trend and raised the threshold of treatment effectiveness to above 90% [8]. Both the European and Spanish consensus reported similar recommendations, with minimal differences in the management of the infection (Table 1).

Our group have been including patients in the European Registry of the Management of *H. pylori* infection (HP-EuReg), an international, multicenter, prospective, non-interventionist registry promoted by the European Helicobacter and Microbiota Study Group, which has already included more than 30,000 patients from over 30 countries [9]. However, studies on *H. pylori* eradication effectiveness in North-East Spain and follow-up on the implementation of consensus guidelines are scarce, but they are clearly needed in order to select the most appropriate treatment or strategy in the management of *H. pylori* infection in each region.

**Table 1:** First-line treatments recommended in Spanish Consensus Conferences (SCC) and Maastricht/Florence Reports during the last decade.

**II** **SCC2005** **[5]**	**III** **SCC2013** **[6]**	**IV** **SCC2016** **[7]**
PCA 7–10 days	PCAM 10 days	PCAM 10–14 days PBMT 10 days
**Maastricht III2007** **[10]**	**Maastricht IV2012** **[11]**	**Maastricht V2016** **[12]**
PC A/M >7 days	PCA 10–14 days PBMT	PCAM 10–14 days PBMT 10–14 days

A: Amoxicillin, B: Bismuth, C: Clarithromycin, M: Metronidazole, P: Proton Pump Inhibitor, T: Tetracycline.

Therefore, the aim of this study was to evaluate the clinical management of *H. pylori* infection in our area of North-East Spain, as well as its appropriateness according to clinical guidelines, and to evaluate eradication treatment effectiveness.

## 2. Results

### 2.1. Baseline Characteristics

A total of 1730 patients were included, approximately 100 patients per year and center. The mean age at diagnosis of *H. pylori* infection was 50.5 ± 15.8 years, and 1035 (59.8%) were women. The age range was 18–90 years, and 1003 patients (58%) were less than 55 years of age. Penicillin allergy was confirmed or suspected in 86 participants (5%). 

The most frequent treatment indication for *H. pylori* eradication was non-investigated dyspepsia or functional dyspepsia (58%), followed by gastric and/or duodenal ulcer (16.1%).

The most widely used diagnostic test was histology (70.1%), followed by urea breath test (28.8%). Only 19 patients (1.1%) were diagnosed using other tests. Histology was the most used test for all treatment indications, with the exception of rosacea, chorioretinopathy and familiar gastric cancer, in which the urea breath test was more frequent; 56% of dyspeptic patients under 55 years (*n* = 614) were diagnosed by histology. The urea breath test was used in 98.6% and histology in 1.4% of patients to confirm *H. pylori* eradication. 

All patients received between one and five lines of treatment, for a total of 2261 eradication treatments recorded. This manuscript focuses on non-penicillin-allergic patients, representing 95% of the sample (1644 patients).

### 2.2. First-Line Prescription Trends

The ‘classic triple’ PCA was the most widely used regimen at the beginning of the decade, with a quick decrease in its use in 2014 and 2015. In 2015 and 2016, it was occasionally prescribed, while its use almost completely disappeared in 2017.

The ‘concomitant’ therapy PCAM’s use started in 2013 and had an exponential increase, becoming the most prescribed therapy in our region in 2015 and 2016 (89%). However, its use has declined in recent years. At the same time, the use of Pylera^®^, which started in 2016, showed remarkable growth, representing half of the prescriptions in 2019.

**Figure 1:**
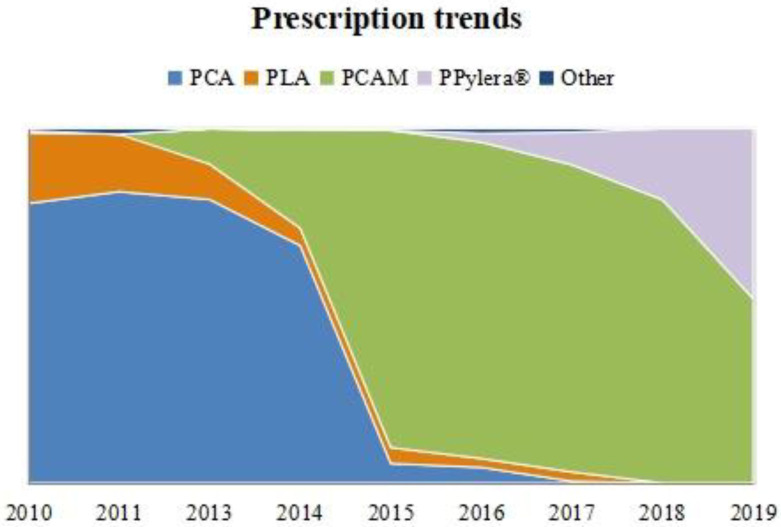
First-line prescription trend over time in non-penicillin-allergic patients. Relative frequencies per year are represented. PCA: PPI + Clarithromycin + Amoxicillin. PCAM: PPI + Clarithromycin + Amoxicillin + Metronidazole. PLA: PPI + Levofloxacin + Amoxicillin. PPylera^®^: PPI + Pylera^®^.

PLA was used as a first-line regimen in 7.5% of cases globally over the decade, especially at the beginning. Prescription trends over time are shown graphically in Figure 1.

Prescription was considered adequate and in accordance with guidelines in 1256 patients (76.4%) and non-adequate in 388 patients (23.6%).

### 2.3. Effectiveness Analysis

#### 2.3.1. First-Line Eradication Rates

In non-penicillin-allergic patients (*n* = 1644), eradication success was achieved in 1162 patients (70.7% by Intention To Treat (ITT)), whereas eradication failure was confirmed in 449 patients (27.3%). Thirty-three patients (2%) did not undergo a confirmation test. The Per Protocol (PP) eradication success rate rose to 72.1%.

**Figure 2:**
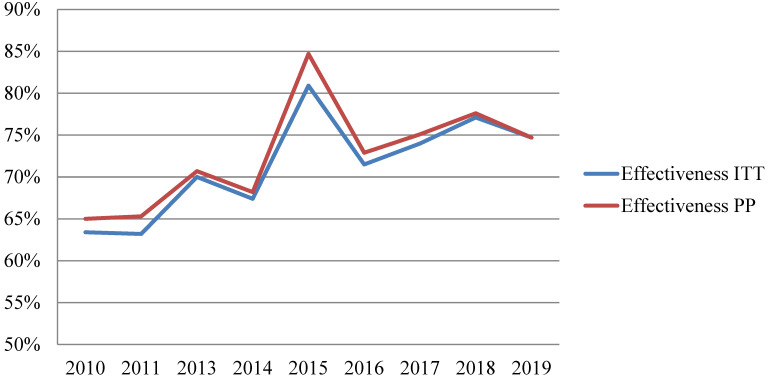
Effectiveness trend of first-line therapy in non-penicillin-allergic patients. ITT: Intention To Treat. PP: Per Protocol.

Overall, an increase in *H. pylori* eradication rates was observed over the decade, rising from 65% at the beginning to 75–80% at the end of this period (Figure 2). Detailed information per year about the effectiveness of each treatment can be found in Supplementary Table S1. 

PCAM showed an effectiveness rate of 76.9% (ITT) in the last decade, but it decreased with time from 84% (ITT) in 2015 to 71% (ITT) in 2016 or 2019. 

PPylera^®^´s global effectiveness was 81.6% (ITT and PP) and no statistically significant differences between PPylera^®^ and PCAM were found (*p* = 0.324 ITT and *p* = 0.551 PP).

The overall effectiveness of PCA was 63.4% (ITT), also changing over time from 59.8% (ITT) in 2010 to 68.8% (ITT) in 2013.

Although the overall effectiveness of the PLA regimen was 69.1% (ITT), higher than the recommended first-line therapy at the moment (PCA), no statistically significant differences between them were found (*p* = 0.225 ITT and *p* = 0.169 PP). Moreover, no statistically significant differences were detected between PLA and PCAM’s global effectiveness (*p* = 0.061 ITT and *p* = 0.051 PP).

The effectiveness of each treatment and the influence of variables other than eradication therapy are shown in Table 2. Treatment-stratified results of these analyses can be seen in Supplementary Tables S2–S5. 

#### 2.3.2. Effectiveness Analysis Based on Other Variables

A longer duration of treatment was associated with greater effectiveness. Univariate logistic regression analysis confirmed a two-fold increased probability of eradication success with 10-day therapies (OR = 2.050; 95%CI: 1.225–3.432) and a 3.5-fold increased probability of eradication success with 14-day treatments (OR = 3.544; 95%CI: 2.026–6.200), compared with 7-day treatments (Table 3). Fourteen-day PCAM therapy showed higher effectiveness compared to 10-day PCAM therapy (84.5%ITT/85.4%PP vs. 78.5% ITT/82.3% PP) but the difference was not statistically significant (*p* = 0.065 ITT and *p* = 0.325 PP) (see Supplementary Table S2).

Only 446 cases had registered detailed PPI prescriptions. A higher effectiveness trend was observed with esomeprazole when compared to omeprazole (82.2% ITT and PP vs. 73.6% ITT/ 74.1% PP), but the difference was not statistically significant (*p* = 0.083 ITT and *p* = 0.102 PP), which was confirmed by univariate logistic regression analysis (OR = 1.655; 95%CI: 0.932–2.937).

There was a statistical association between treatment effectiveness and sex: the effectiveness in males was higher (5.3% ITT and 6.2% PP) than in females. Univariate analysis confirmed this finding, with the success probability being 29.9% higher in males (OR = 1.299; 95% CI: 1.044–1.617). No significant differences in effectiveness by recruiting center were found. 

Although the overall analysis did not find an association between treatment effectiveness and age, treatment-stratified analysis showed this association for PCAM and PLA use. (Supplementary Tables S2 and S4). PCAM had 7.7% increased effectiveness in patients ≥55 years compared to <55 years of age, and treatment-stratified multivariate analysis found an increase of 47.2% in success probability in patients being ≥55 years old (OR = 1.472; 95%CI: 1.020–2.126). Effectiveness of PLA in patients <55 years of age was 18.6% higher (*p* = 0.027 ITT and *p* = 0.018 PP) vs. older patients, and treatment-stratified multivariate analysis confirmed the 66% lower effectiveness in patients ≥55 years of age (OR = 0.340; CI95%: 0.143–0.812).

We also compared the effectiveness and appropriateness of prescription. Treatment effectiveness increased by 6.5% (ITT) and 6.8% (PP) if the recommended therapy was used (*p* = 0.014 ITT and *p* = 0.010 PP). Univariate logistic regression obtained 33.5% increased success probability in patients treated with the recommended regimen (OR = 1.355; 95% IC: 1.063–1.729) (Table 3).

As previously described, the univariate analysis performed with the first-line treatment showed that effectiveness was associated with the following variables: extended therapies (10-day (OR = 2.050; 95% CI: 1.225–3.432) or 14-day length (OR = 3.544; 95% CI: 2.026–6.200)), male gender (OR = 1.299; 95% CI: 1.044–1.617) and appropriateness (OR = 1.355; IC95% CI: 1.063–1.729). Differences by treatment type were also observed (see Table 3), but only the variables of treatment type and sex maintained statistical significance in the multivariate analysis, with the effectiveness in males being 27% higher than in females (OR = 1.270; 95% CI: 1.013–1.592).

## 3. Discussion

*Helicobacter pylori* is the most frequent infection in humans, present in almost 50% of the world population [1], sometimes associated with important symptoms and consequences. In this study, we included 1644 patients non-allergic to penicillin, from two areas of the Regional Health System, between 2010 and 2019 for the purpose of monitoring the management and results of *H. pylori* infection, to improve the daily clinical practice.

More than 50% of patients were women, a fact that contrasts with the known higher prevalence of the infection in men (43.2% vs. 37.6%) [13]. This could be explained because the most frequent indication of *H. pylori* treatment was dyspepsia, a pathology more common in women [14].

Comparing our data with those recently published by the Hp-EuReg [4], the proportion of women in both studies was similar (59.8%). Moreover, the mean age (around 50 years) and the proportion of *H. pylori* treatments due to peptic ulcer disease (16%) were very similar. However, in our study, the proportion of *H. pylori* treatments because of dyspepsia was lower (58% vs. 83%). This difference could be related to differences in the classification of *H. pylori* eradication indications or to the treatment of pathologies such as chorioretinopathy or rosacea in our centers, which were related to the infection in the past, and are excluded now from most clinical guidelines [6,11].

Endoscopy with histology followed by urea breath test were the most widely diagnostic tests used. Both tests are recommended by guidelines for the diagnosis of *H. pylori* infection and the investigation of dyspepsia, due to their accessibility and price. We detected high use of endoscopy in dyspeptic patients under 55 years of age with no symptoms or alarm signs, when the Test and Treat strategy with non-invasive diagnostic methods is recommended in these circumstances [15]. This finding is important and shows the need to implement new actions to improve the management of these patients according to current guidelines.

Increased effectiveness was observed when the prescription followed the recommendations of guidelines, becoming another important factor that could improve *H. pylori* eradication rates in daily clinical practice. Three out of four (76.4%) of the prescribed treatments followed the clinical guidelines at the time of the prescription or within the six-month period following the publication of new guidelines, a time period that we considered enough to adapt the clinical management to the new clinical guidelines in a specialized gastroenterological environment. 

The overall effectiveness of first-line therapies in patients non-allergic to penicillin was 70.7% by ITT, a worrying rate far from the target of 90%. Statistical analysis found higher effectiveness in the case of quadruple therapies, longer treatments, males and adequate adherence to clinical guidelines. However, stratified analysis by treatment showed some notable facts, which are detailed below.

### 3.1. PCA

The triple therapy PCA has been recommended since the beginning of the infection in the 1990s. In our sample, it was the most prescribed therapy at the beginning of the decade, declining in use in 2014, after the publication of the third SCC in 2013, when more effective therapies were proposed [6]. Its prescription continued declining until 2017, when it had nearly disappeared. This trend was according to the main current recommendations [7,12]. 

PCA effectiveness is known to be insufficient. A Spanish meta-analysis of 32 studies performed between 2007 and 2008 showed an eradication rate of 80% ITT [16]. Other Spanish data reported rates of 71% [17], always less than 75% [18]. Local results, all before the year 2000, showed eradication rates between 77.4% and 89.2% [19,20]. We obtained a lower PCA effectiveness rate (63.4% ITT). Eradication rates with this therapy were enhanced by 7% when the duration was increased from 7 to 10 days, a fact already known from other publications [21], but without statistical significance in our study. Prior antibiotic treatments (especially for urinary tract infection) increase resistance and decrease effectiveness rates in women [22,23]. In our area, the effectiveness of PCA was 7% higher in men than in women, but multivariate analysis stratified by treatment could not confirm this influence.

### 3.2. PCAM

Nowadays, the prescription of the ‘concomitant’ therapy PCAM is growing and it is recommended as the empirical first-line therapy in current clinical guidelines [8,12]. After the publication of the third SCC [6], its use increased, and after 2015, it became the most prescribed treatment in our region. However, we observed a decrease in the prescription of PCAM from 2016 onwards, when Pylera^®^ was launched in our country.

The overall effectiveness rate was 76.9% ITT in the last decade, with changes depending on the year (84.8% in 2015 or 70.9% in 2016 and 2019), higher than the standard triple therapy but lower than other reported studies, with rates around 89% [21,24,25,26]. However, retrospective clinical practice studies in Turkey and Korea found similar rates (around 75–79%) to those found in our study [27,28]. The effectiveness of PCAM was not superior to PPylera^®^ or PLA, which is consistent with other publications [29,30].

Although most prescriptions were of 10-day duration, an increase of 6% in effectiveness was observed when 14-day prescriptions were used (78.5% ITT vs. 84.5% ITT, respectively). The improvement in the effectiveness by increasing the duration was already known [21,26,31,32], but statistical significance was not reached in our sample, so the duration of the prescription should be individualized. Again, the effectiveness of a clarithromycin-based therapy was higher in men (80.1% ITT vs. 74.9% ITT) [22,23], but it was not statistically significant.

Multivariate analysis stratified by treatment confirmed the higher effectiveness of PCAM in patients ≥ 55 years compared to patients under 55 years. This finding could be due to worse adherence in the younger group (it was not registered in our study). However, there are studies that could not associate adherence with demographic variables [33], and another study found that young patients, under the age of 30, were the most compliant [34].

### 3.3. PBMT–PPYLERA^®^

Bismuth-containing quadruple therapy PBMT was traditionally recommended as rescue therapy in patients non-allergic to penicillin, or as a first-line treatment in allergic patients. However, in the latest clinical guidelines, it was proposed as the first empirical line to all patients as an alternative to PCAM, pending confirmation of its effectiveness in our area at the time of publication [7]. Maastricht V recommended its use in the case of local dual resistance to clarithromycin and metronidazole [12].

The use in our area began after its commercialization in 2016 and its recommendation in the fourth SCC, progressively increasing until the end of the decade, being used in 48% of *H. pylori* eradication regimens in 2019.

We observed that PPylera^®^ (effectiveness 81.6%) had the highest effectiveness together with PCAM, without statistical differences between them. Therefore, we can confirm its effectiveness in our area. This effectiveness is similar to results reported by Agudo-Fernández et al., another Spanish real-life study performed in 2018 (78.15% ITT) [35], but lower than others published in our region (85.9% ITT) [30], or other Spanish areas with rates over 90% [36,37].

### 3.4. PLA

Fear of creating or even increasing quinolone resistance is one of the reasons for not recommending PLA as first-line treatment. Although it has never been proposed as a first line by clinical guidelines, it was chosen by physicians in 7.5% of the cases, most of them at the beginning of the decade. In fact, in one center, PLA represented 41.3% of the prescriptions in 2010. We consider it important to emphasize that these incorrect prescriptions were scheduled by the same physicians, active in clinical consultations at the beginning of the decade, with many years of practice, who were probably less flexible in adopting new recommendations in their clinical practice.

Although its effectiveness as second-line therapy is widely known, there are few studies evaluating its effectiveness as a first line. In 2007, Gisbert et al. published eradication rates of 84–88% [38]. Another author published high rates between 86% and 93% [39,40,41]. However, two Chinese systematic reviews showed rates around 80% and less than 75% when assessing studies after 2012 [42,43]. We report here an eradication rate of 69.1% ITT and 70.8% PP, higher than the most prescribed therapy at the beginning of the decade, PCA, but without a significant difference. 

Multivariate analysis stratified by treatment confirmed the higher effectiveness of PLA in patients <55 years compared to patients over 55 years. This finding could be explained by a higher previous intake of antibiotics in older people and, therefore, probably higher resistance rates in these patients.

### 3.5. Strengths and Weaknesses

The large sample size and the long period of time analyzed depict accurately the management of *H. pylori* infection in our area. Indeed, comparison with current guidelines allows us to monitor and improve clinical practice based on an evidence-based quality standard. However, results should be interpreted with caution given the retrospective design of the study, although it has the advantage of not introducing any bias, which may be present when participants in a prospective study know that they are being monitored.

## 4. Materials and Methods

### 4.1. Study Design

This is a retrospective, observational study assessing the management of *H. pylori* infection between January 2010 and December 2019. This is a parallel extension of the sub-analysis from our cases included in the European Registry on *Helicobacter Pylori* management (HP-EuReg), an international, multicenter, non-interventional registry conducted by the European Helicobacter and Microbiota Study Group. Cases were not included in 2012 because an interventional study in one of the centers was conducted that year.

The study was conducted in the outpatient units of two defined areas of the Regional Health System in Aragón (“Lozano Blesa” University Hospital of Zaragoza and “Obispo Polanco” Hospital of Teruel, Spain). These two centers are the reference for a total population of approximately 350,000 people and 38 primary care centers and reflect the usual clinical practice at a gastroenterologist specialist level.

Inclusion criteria were age ≥ 18 years, current *H. pylori* infection and absence of previous eradication treatment (*naïve*). Patients were excluded in case of eradication treatment prior to 2010 (not *naïve*) and a lack of accurate demographic or treatment data. 

The initial diagnosis of *H. pylori* infection was made based on a positive result of any of the following diagnostic tests: urea breath test with ^13^CO ≥ 2.5‰ (UBTest^®^, Otsuka Pharmaceutical, Tokyo, Japan), serology, histochemistry in gastric samples or stool antigen test. To confirm eradication, urea breath test with ^13^CO ≥ 2.5‰ and histology were used.

### 4.2. Variables

Medical records were reviewed to collect the following variables: center of origin, prescription date, age, age group, sex, penicillin allergy, prescribed antibiotic regimen and duration, Proton Pump Inhibitor (PPI) treatment, agreement with current clinical practice guidelines and effectiveness. The variable age group had two categories (patients under 55 years of age, and patients with 55 years of age or more). We chose this cut-off of 55 years as it is the most common age threshold for performing endoscopy in uninvestigated dyspepsia.

Effectiveness had 2 categories: success (negative eradication confirmation test) or failure (positive eradication confirmation test).

Data were saved in an Access database specifically designed for this purpose (Microsoft Office Access 2017, Microsoft Corporation, Washington, DC, USA).

### 4.3. Definition of Agreement with Guidelines

First-line treatment in patients non-allergic to penicillin was considered appropriate if it followed the recommendation of the SCC at the time of the prescription. When a new guideline had just been published, it was considered appropriate to follow the previous guidelines during the first 6 months, as a period of adaptation to the new recommendations:

- First triene (2010–August 2013, and 6 months after the III (third) Spanish Consensus Conference (SCC) publication): PCA (PPI + Clarithromycin + Amoxicillin);
- Second triene (February 2013–November 2016, and 6 months after the IV SCC publication): PCAM(PPI + Clarithromycin + Amoxicillin + Metronidazole);
- Third triene (May 2016–December 2019): PPylera ^®^ (PPI + Pylera^®^, the three-in-one capsule containing Bismuth Subcitrate + Metronidazole + Tetracycline).

### 4.4. Statistical Analysis and Ethics Statement

Qualitative variables were presented as absolute (frequency) and relative (%) values. Normality of quantitative variables was assessed by Kolmogorov–Smirnov test and they were presented as mean and standard deviation (SD). Moreover, graphic representations were added.

A bivariate analysis between efficacy and the different variables (sex, age group, center, PPI, antibiotic regimen, appropriateness and duration) was performed. The relationship between qualitative variables was performed by the Chi Square test. Univariate and multivariate analysis between efficacy and the different variables was performed using binary logistic regression, with OR as an effect measure and a 95% Confidence Interval (CI); PCAM was chosen as the reference category in the treatment analysis as it was the most frequent treatment in the sample.

Efficacy analysis was performed by Intention To Treat (ITT: considering failure cases of loss to follow-up, which means no confirmatory test during the 12 months after a treatment) and Per Protocol (PP: including only patients who have completed follow-up). A *p*-value < 0.05 was considered statistically significant.

Sample size was estimated to assess the efficacy of treatments (main objective) using the Epidat 4.2 program (Dirección General de Innovación y Gestión de la SaludPública, Xunta de Galicia (Spain), Organización Panamericana de la Salud (OPS-OMS), Instituto Superior de Ciencias Médicas de La Habana (Cuba)). It was based on an initial analysis of data from 2010 and 2011, finding an overall treatment efficacy of 64%. For a confidence level of 95% and a precision of 3%, a sample size of 984 patients was estimated.

The study was conducted according to the guidelines of the Declaration of Helsinki, and authorized by the two hospitals. The Hp-EuReg protocol was approved by the Ethics Committee of La Princesa University Hospital (Madrid, Spain) and was registered at ClinicalTrials.gov (code NCT02328131). The full protocol includes more details [9].

## 5. Conclusions

Although *H. pylori* infection management should be individualized in daily clinical practice, we found an overuse of upper endoscopy procedures in dyspeptic patients < 55 years, inadequate drug prescription in 23.6% of the cases, use of H. pylori treatments not included in clinical guidelines (such as PLA as first-line therapy) and the treatment of patients without indication. All these findings should be considered for the implementation of action to improve the correct clinical management of *H. pylori* infection.

Triple therapies should not be prescribed because quadruple therapies achieve the highest eradication rates. However, despite the use of quadruple therapies and optimized treatments (longer duration and using esomeprazol), the effectiveness in daily clinical practice is still lower than expected, and far from the target of 90%. PLA therapy was more effective in patients under 55 years, and PCAM was more effective in patients over 55 years, which implies that age may be a variable to consider before prescribing any treatment. 

## Figures and Tables

**Table 2:** Effectiveness of 1644 first-line treatments in non-penicillin-allergic patients: detailed analysis by variables.

Variables	Categories	ITT			PP		
		N	%	95% CI	N	%	95% CI
Treatment	PCAM	737	76.9%	73.8–79.8	719	78,9%	75.7–81.7
	PCA	686	63.4%	59.7–66.9	676	64.3%	60.7–67.9
	PLA	123	69.1%	60.5–76.6	120	70.8%	62.2–78.2
	PPylera^®^	87	81.6%	72.2–88.4	87	81.6%	72.2–88.4
	Other treatments	11	36.4%	15.2–64.6	9	44.4%	18.9–73.3
	*p*-value	**<0.001**			**<0.001**		
Duration	7 days	67	56.7%	44.8–67.9	66	57.6%	45.6–68.8
	10 days	612	72.9%	69.2–76.2	598	74.6%	70.9–77.9
	14 days	333	82.3%	77.8–86.0	329	83.3%	78.9–86.9
	*p*-value	**<0.001**			**<0.001**		
PPI	Omeprazole	284	73.6%	68.2–78.4	282	74.1%	68.7–78.9
	Esomeprazole	101	82.2%	73.6–88.4	101	82.2%	73.6–88.4
	Other PPI	61	70.5%	58.1–80.4	61	70.5%	58.1–80.4
	*p*-value	0.154			0.169		
Sex	Female	971	68.5%	65.6–71.3	955	69.6%	66.6–72.5
	Male	673	73.8%	70.4–77.0	656	75.8%	72.3–78.9
	*p*-value	**0.019**			**0.007**		
Center	Center 1 (Z)	766	70.5%	67.2–73.6	744	72.6%	69.3–75.7
	Center 2 (T)	878	70.8%	67.8–73.8	867	71.7%	68.7–74.6
	*p*-value	0.878			0.708		
Age	<55 years	962	69.6%	66.7–72.5	938	71.4%	68.5–74.2
	≥55 years	682	72.1%	68.7–75.4	673	73.1%	69.6–76.3
	*p*-value	0.274			0.459		
Appropriateness	No	388	65.7%	60.9–70.3	381	66.9%	62.1–71.5
	Yes	1256	72.2%	69.7–74.6	1230	73.7%	71.2–76.1
	*p*-value	**0.014**			**0.010**		
Total		1644	70.7%	68.4–72.8	1611	72.1%	69.9–74.3

ITT: Intention To Treat. PP: Per Protocol. N: total of patients included. %: proportion of patients presenting effectiveness. 95% CI: Confidence Interval. PCA: PPI + Clarithromycin + Amoxicillin PLA: PPI + Levofloxacin + Amoxicillin. PCAM: PPI + Clarithromycin + Amoxicillin + Metronidazole. PPylera^®^: single capsule bismuth quadruple therapy (PPI + Pylera^®^). PPI: Proton Pump Inhibitor.Center 1 (Z): Lozano Blesa University Hospital, Zaragoza (Spain). Center 2 (T): Obispo Polanco Hospital, Teruel (Spain). Bold values denote statistical significance at the *p*-value < 0.05 level. *p*-value: Chi Square test for proportion difference was applied.

**Table 3:** Analysis of association with global efficacy by univariate and multivariate binary logistic regression. Intention To Treat (ITT) analysis of 1644 first-line treatments in non-penicillin-allergic patients.

		Univariate		Multivariate	
		OR (95% CI)	*p*-Value	OR (95% CI)	*p*-Value
Treatment	[R: PCAM]	1		1	
	PCA	0.520 (0.412–0.655)	**<0.001**	0.489 (0.376–0.636)	**<0.001**
	PLA	0.671 (0.441–1.020)	0.062	0.595 (0.305–1.014)	0.056
	PPylera^®^	1.330 (0.753–2.350)	**0.325**	1.266 (0.714–2.243)	0.420
	Othertreatments	0.171 (0.050–0.592)	**0.005**	0.162 (0.05–0.587)	**0.006**
Duration	[R: 7 days]	1		-	-
	10 days	2.050 (1.225–3.432)	**0.006**		
	14 days	3.544 (2.026–6.200)	**<0.001**	-	-
PPI	[R: Omeprazole]	1			
	Esomeprazole	1.655 (0.932–2.937)	0.085	-	-
	Other PPI	0.857 (0.466–1.578)	0.621	-	-
Sex	[R: Female]	1		1	
	Male	1.299 (1.044–1.617)	**0.021**	1.270 (1.013–1.592)	**0.038**
Center	[R: Center 1 (Z)]	1		1	
	Center 2 (T)	1.017 (0.822–1.258)	0.878	1.132 (0.902–1.419)	0.284
Age	[R: <55 years]				
	≥55 years	1.129 (0.090–1.401)	0.274	1.106 (0.885–1.383	0.374
Appropriateness	[R: No]	1		1	
	Yes	1.355 (1.063–1.729)	**0.014**	0.893 (0.645–1.236)	0.493

Effect size expressed as OR (Odds Ratio) and 95% CI (Confidence Interval) and performed using binary logistic regression models (dependent variable: ITT effectiveness). Multivariate model adjusted by treatment, sex, center, age, appropriateness and treatment indication. R: category of reference used for the logistic regression. PCAM: PPI + Clarithromycin + Amoxicillin + Metronidazole. PCA: PPI + Clarithromycin + Amoxicillin. PLA: PPI + Levofloxacin + Amoxicillin. PPylera^®^: single capsule bismuth quadruple therapy (PPI + Pylera^®^). PPI: Proton Pump Inhibitor. Center 1 (Z): Lozano Blesa University Hospital, Zaragoza (Spain). Center 2 (T): Obispo Polanco Hospital, Teruel (Spain). Bold values denote statistical significance at the *p*-value < 0.05 level.

## Data Availability

The data presented in this study are available on request to the corresponding author.

## Supplementary Materials

The following are available online at https://www.mdpi.com/article/10.3390/antibiotics11050698/s1, Table S1: Effectiveness detailed by prescription year in non-allergic to penicillin patients, overall effectiveness and disaggregated by first-line therapy. Table S2: Effectiveness of first-line treatments with PCAM (PPI + Clarithromycin + Amoxicillin + Metronidazole) in non-penicillin-allergic patients: detailed analysis by variables. Table S3. Effectiveness of first-line treatments with PCA (PPI + Clarithromycin + Amoxicillin) in non-penicillin-allergic patients: detailed analysis by variables. Table S4. Effectiveness of first-line treatments with PLA (PPI + Levofloxacin + Amoxicillin) in non-penicillin-allergic patients: detailed analysis by variables. Table S5. Effectiveness of first-line treatments with PPylera^®^ (PPI + Pylera^®^) in non-penicillin-allergic patients: detailed analysis by variables.
