# Supplementary material for: Management of Helicobacter Pylori Infection and Effectiveness Rates in Daily Clinical Practice in Spain: 2010–2019
Source: Antibiotics (Basel). 2022 May 20;11(5):698. doi: 10.3390/antibiotics11050698 (PMC9171584; doi:10.3390/antibiotics11050698)
Supplement: Supplementary file 1 [file antibiotics-11-00698-s001.zip › antibiotics-1679193-supplementary.pdf]

Supplementary Materials

Table S1. Effectiveness detailed by prescription year in non-allergic to penicillin patients, overall effectiveness and disaggregated by first line therapy.

Anual effectiveness		TOTAL n=1644	PCA n=686	PLA n=123	PCAM n=737	PPylera® n=87	Other n=11
2010	ITT	102/161<br>63.4%	76 /127<br>59.8%	25 /32<br>78.1%	1 /1<br>100%	-	-
	PP	102/157<br>65%	76/125<br>60.8%	25/31<br>80.6%	1/1<br>100%	-	-
2011	ITT	156/247<br>63.2%	124/203<br>61.1%	31/40<br>77.5%			1/4<br>25%
	PP	156/239<br>65.3%	124/198<br>62.6%	31/38<br>81.6%	-	-	1/3<br>33.3%
2013	ITT	140/200<br>70%	110/160<br>68.8%	12/20<br>60%	18/20<br>90%		
	PP	140/198<br>70.7%	110/159<br>69.2%	12/20<br>60%	18/19<br>94.7%	-	-
2014	ITT	178/264<br>67.4%	115/177<br>65%	5/13<br>38.5%	57/73<br>78.1%		1/1<br>100%
	PP	178/261<br>68.2%	115/176<br>65.3%	5/13<br>38.5%	57/71<br>80.3%	-	
2015	ITT	161/199<br>80.9%	4/11<br>36.4%	6/9<br>66.7%	151/178<br>84.8%		0/1<br>0%
	PP	161/190<br>84.7%	4/11<br>36.4%	6/9<br>66.7%	151/169<br>89.3%	-	0/1<br>0%
2016	ITT	113/158<br>71.5%	6/7<br>85.7%	3/4<br>75%	100/141<br>70.9%	4/4<br>100%	0/2<br>0%
	PP	113/155<br>72.9%	6/7<br>85.7%	3/4<br>75%	100/138<br>72.5%	4/4<br>100%	0/2<br>0%
2017	ITT	145/196<br>74%	0/1<br>0%	3/5<br>60%	125/170<br>73.5%	15/18<br>83.3%	2/2<br>100%
	PP	145/193<br>75.1%	-	3/5<br>60%	125/168<br>74.4%	15/18<br>83.3%	2/2<br>100%
2018	ITT	111/144	-	-	87/115	24/29	-

	**77.1%**			**75.7%**	**82.8%**	
**PP**	**111/143**<br>**77.6%**	**-**	**-**	**87/114**<br>**76.3%**	**24/29**<br>**82.8%**	**-**
**2019 ITT**	**56/75**<br>**74.7%**	**-**	**-**	**28/39**<br>**71.8%**	**28/36**<br>**77.8%**	**-**
**PP**	**56/75**<br>**74.7%**	**-**	**-**	**28/39**<br>**71.8%**	**28/36**<br>**77.8%**	**-**

Expressed as number of successful eradications and percentage: n (%). ITT: Intention To Treat. PCA: classic triple therapy (PPI + Clarithromycin + Amoxicillin). PCAM: concomitant non-bismuth quadruple therapy (PPI + Clarithromycin + Amoxicillin + Metronidazole). PLA: levofloxacin based triple therapy (PPI + Levofloxacin + Amoxicillin). PP: Per Protocol. PPylera®: single capsule bismuth quadruple therapy (PPI + Pylera®). PPI: Proton Pump Inhibitor.

Table S2. Effectiveness of first line treatments with PCAM (PPI + Clarithromycin + Amoxicillin + Metronidazole) in non-penicillin-allergic patients: detailed analysis by variables.

**PCAM**	**Categories**	**ITT**			**PP**		
		**N**	**%**	**95% CI**	**N**	**%**	**95% CI**
**Duration**	**7 days**	0	-	-	0	-	-
	**10 days**	260	78.5%	73.1-83.0	248	82.3%	77.0-86.5
	**14 days**	297	84.5%	80.0-88.2	294	85.4%	80.9-89.0
	p-value	0.065			0.325		
**PPI**	**Omeprazole**	68	86.8%	76.7-92.9	67	88.1%	78.2-93.8
	**Esomeprazole**	74	89.2%	80.1-94.4	74	89.2%	80.1-94.4
	**Other PPI**	22	81.8%	61.5-92.7	22	81.8%	61.5-92.7
	p-value	0.656			0.648		
**Sex**	**Female**	451	74.9%	70.7-78.7	440	76.8%	72.7-80.5
	**Male**	286	80.1%	75.1-84.3	279	82.1%	77.2-86.1
	p-value	0.107			0.092		
**Center**	**Center 1 (Z)**	381	77.7%	73.2-81.6	371	79.8%	75.4-83.6
	**Center 2 (T)**	356	76.1%	71.4-80.3	348	77.9%	73.2-81.9
	p-value	0.614			0.531		
**Age**	**<55 years**	435	73.8%	69.5-77.7	421	76.2%	72.0-80.1
	**≥55 years**	302	81.5%	76.7-85.4	298	82.6%	77.8-86.4
	p-value	**0.015**			**0.041**		
**Appropriateness**	**No**	1	100.0%	-	1	100.0%	-
	**Yes**	736	76.9%	73,7-79,8	718	78.8%	75,7-81,7
	p-value	0.584			0.604		
**Total**		737	76.9%	73.8-79.8	719	78.9%	75.7-81.7

PCAM: PPI + Clarithromycin + Amoxicillin + Metronidazole. PPI: Proton Pump Inhibitor. ITT: Intention To Treat. PP: Per Protocol. N: total of patients included. %: proportion of patients presenting effectiveness. 95%CI: Confidence Interval. Center 1 (Z): Lozano Blesa University Hospital, Zaragoza (Spain). Center 2 (T): Obispo Polanco Hospital, Teruel (Spain). Bold values denote statistical significance at the *p-value* < 0.05 level. p-value: Chi Square test for proportion difference was applied.

Table S3. Effectiveness of first line treatments with PCA (PPI + Clarithromycin + Amoxicillin) in non-penicillin-allergic patients: detailed analysis by variables.

PCA	Categories	ITT			PP		
		N	%	95% CI	N	%	95% CI
**Duration**	**7 days**	62	58.1%	45.7-69.5	61	59.0%	46.5-70.5
	**10 days**	248	64.9%	58.8-70.6	247	65.2%	59.0-70.8
	**14 days**	31	58.1%	40.8-73.6	30	60.0%	42.3-75.4
	p-value	0.506			0.610		
**PPI**	**Omeprazole**	199	69.8%	63.1-75.8	199	69.8%	63.1-75.8
	**Esomeprazole**	16	56.3%	33.2-76.9	16	56.3%	33.2-76.9
	**Other PPI**	34	61.8%	45.0-76.1	34	61.8%	45.0-76.1
	p-value	0.381			0.381		
**Sex**	**Female**	399	60.7%	55.8-65.3	396	61.1%	56.2-65.8
	**Male**	287	67.2%	61.6-72.4	280	68.9%	63.3-74.1
	p-value	0.077			**0.037**		
**Center**	**Center 1 (Z)**	258	59.7%	53.6-65.5	250	61.6%	55.4-67.4
	**Center 2 (T)**	428	65.7%	61.0-70.0	426	66.0%	61.3-70.3
	p-value	0.116			0.253		
**Age**	**<55 years**	406	63.1%	58.3-67.6	400	64.0%	59.2-68.6
	**≥55 years**	280	63.9%	58.2-69.3	276	64.9%	59.1-70.2
	p-value	0.815			0.820		
**Appropriateness**	**No**	253	65.2%	59.2-70.8	251	65.7%	59.7-71.3
	**Yes**	433	62.4%	57.7-66.8	425	63.5%	58.9-68.0
	p-value	0.453			0.563		
**Total**		686	63.4%	59.7-66.9	676	64.3%	60.7-67.9

PCA: PPI + Clarithromycin + Amoxicillin. PPI: Proton Pump Inhibitor. ITT: Intention To Treat. PP: Per Protocol. N: total of patients included. %: proportion of patients presenting effectiveness. 95%CI: Confidence Interval. Center 1 (Z): Lozano Blesa University Hospital, Zaragoza (Spain). Center 2 (T): Obispo Polanco Hospital, Teruel (Spain). Bold values denote statistical significance at the *p-value* < 0.05 level. p-value: Chi Square test for proportion difference was applied.

Table S4. Effectiveness of first line treatments with PLA (PPI + Levofloxacin + Amoxicillin) in non-penicillin-allergic patients: detailed analysis by variables.

PLA	Categories	ITT			PP		
		**N**	**%**	**95% CI**	**N**	**%**	**95% CI**
**Duration**	**7 days**	4	50.0%	15.0-85.0	4	50.0%	15.0-85.0
	**10 days**	39	66.7%	51.0-79.4	38	68.4%	52.5-80.9
	**14 days**	1	100.0%	-	1	100.0%	-
	p-value	0.613			0.591		
**PPI**	**Omeprazole**	14	57.1%	32.6-78.6	13	61.5%	35.5-82.3
	**Esomeprazole**	7	71.4%	35.9-91.8	7	71.4%	35.9-91.8
	**Other PPI**	5	80.0%	37.6-96.4	5	80.0%	37.6-96.4
	p-value	0.605			0.734		
**Sex**	**Female**	65	69.2%	57.2-79.1	65	69.2%	57.2-79.1
	**Male**	58	69.0%	56.2-79.4	55	72.7%	59.8-82.7
	p-value	0.975			0.675		
**Center**	**Center 1 (Z)**	88	68.2%	57.9-77.0	86	69.8%	59.4-78.5
	**Center 2 (T)**	35	71.4%	54.9-83.7	34	73.5%	56.9-85.4
	p-value	0.725			0.683		
**Age**	**<55 years**	70	77.1%	66.0-85.4	68	79.4%	68.4-87.3
	**≥55 years**	53	58.5%	45.1-70.7	52	59.6%	46.1-71.8
	p-value	**0.027**			**0.018**		
**Appropriateness**	**No**	123	69.1%	60.5-76.6	120	70.8%	62.2-78.2
	**Yes**	0	-	-	0	-	-
	p-value	-			-		
**Total**		123	69.1%	60.5-76.6	120	70.8%	62.2-78.2

PLA: PPI + Levofloxacin + Amoxicillin. PPI: Proton Pump Inhibitor. ITT: Intention To Treat. PP: Per Protocol. N: total of patients included. %: proportion of patients presenting effectiveness. 95%CI: Confidence Interval. Center 1 (Z): Lozano Blesa University Hospital, Zaragoza (Spain). Center 2 (T): Obispo Polanco Hospital, Teruel (Spain). Bold values denote statistical significance at the *p-value* < 0.05 level. p-value: Chi Square test for proportion difference was applied.

Table S5. Effectiveness of first line treatments with PPylera®(PPI + Pylera®). in non-penicillin-allergic patients: detailed analysis by variables.

PPylera®	Categories	ITT			PP		
		N	%	95% CI	N	%	95% CI
**Duration**	**7 days**	0	-	-	0	-	-
	**10 days**	64	84.4%	73.6-91.3	64	84.4%	73.6-91.3
	**14 days**	2	100.0%	34.2-100.0	2	100.0%	34.2-100.0
	p-value	0.544			0.544		
**PPI**	**Omeprazole**	3	100.0%	43.9-100.0	3	100.0	43.9-100.0
	**Esomeprazole**	2	100.0%	34.2-100.0	2	100.0%	34.2-100.0
	**Other PPI**	0	-	-	0	-	-
	p-value	-			-		
**Sex**	**Female**	48	75.0%	61.2-85.1	48	75.0%	61.2-85.1
	**Male**	39	89.7%	76.4-95.9	39	89.7%	76.4-95.9
	p-value	0.078			0.078		
**Center**	**Center 1 (Z)**	31	90.3%	75.1-96.7	31	90.3%	75.1-96.7
	**Center 2 (T)**	56	76.8%	64.2-85.9	56	76.8%	64.2-85.9
	p-value	0.119			0.119		
**Age**	**<55 years**	44	86.4%	73.3-93.6	44	86.4%	73.3-93.6
	**≥55 years**	43	76.7%	62.3-86.8	43	76.7%	62.3-86.8
	p-value	0.247			0.247		
**Appropriateness**	**No**	0	-	-	0	-	-
	**Yes**	87	81.6%	72.2-88.4	87	81.6%	72.2-88.4
	p-value	-			-		
**Total**		87	81.6%	72.2-88.4	87	81.6%	72.2-88.4

PPylera®: single capsule bismuth quadruple therapy (PPI + Pylera®). PPI: Proton Pump Inhibitor. ITT: Intention To Treat. PP: Per Protocol. N: total of patients included. %: proportion of patients presenting effectiveness. 95%CI: Confidence Interval. Center 1 (Z): Lozano Blesa University Hospital, Zaragoza (Spain). Center 2 (T): Obispo Polanco Hospital, Teruel (Spain). Bold values denote statistical significance at the *p-value* < 0.05 level. p-value: Chi Square test for proportion difference was applied.
